# Validation of Different Combination of Three Reversing Half-Hitches Alternating Posts (RHAPs) Effects on Arthroscopic Knot Integrity

**Published:** 2017-05-15

**Authors:** Alexander CM. Chong, Daniel J. Prohaska, Brian P. Bye

**Affiliations:** 1 Via Christi Health, Department of Graduate Medical Education, Wichita, KS; 2 University of Kansas School of Medicine-Wichita, Department of Orthopaedics, Wichita, KS; 3 Advanced Orthopaedics Associates, Wichita, KS

**Keywords:** arthroscopy, suture techniques, orthopedics

## Abstract

**Introduction:**

With arthroscopic techniques being used, the importance of knot tying has been examined. Previous literature has examined the use of reversing half-hitches on alternating posts (RHAPs) on knot security. Separately, there has been research regarding different suture materials commonly used in the operating room. The specific aim of this study was to validate the effect of different stacked half-hitch configuration and different braided suture materials on arthroscopic knot integrity.

**Methods:**

Three different suture materials tied with five different RHAPs in arthroscopic knots were compared. A single load-to-failure test was performed and the mean ultimate clinical failure load was obtained.

**Results:**

Significant knot holding strength improvement was found when one half-hitch was reversed as compared to baseline knot. When two of the half-hitches were reversed, there was a greater improvement with all knots having a mean ultimate clinical failure load greater than 150 newtons (N). Comparison of the suture materials demonstrated a higher mean ultimate clinical failure load when Force Fiber^®^ was used and at least one half-hitch was reversed. Knots tied with either Force Fiber^®^ or Orthocord^®^ showed 0% chance of knot slippage while knots tied with FiberWire^®^ or braided fishing line had about 10 and 30% knot slippage chances, respectively.

**Conclusions:**

A significant effect was observed in regards to both stacked half-hitch configuration and suture materials used on knot loop and knot security. Caution should be used with tying three RHAPs in arthroscopic surgery, particularly with a standard knot pusher and arthroscopic cannulas. The findings of this study indicated the importance of three RHAPs in performing arthroscopic knot tying and provided evidence regarding discrepancies of maximum clinical failure loads observed between orthopaedic surgeons, thereby leading to better surgical outcomes in the future.

## Introduction

Rotator cuff tears are common, potentially leading to shoulder pain and dysfunction. During upper extremity movement (especially throwing and swimming), the anterior aspect of the supraspinatus tendon is under the greatest load and commonly is involved in rotator cuff injury.^1^ Arthroscopic techniques are used for rotator cuff repairs to minimize invasiveness and pain. During arthroscopic surgery, the surgeon commonly is required to tie a sliding arthroscopic knot followed by a series of reversing half-hitches on alternating posts (RHAPs) in an attempt to yield a knot capable of secure tissue fixation. This fixation also must be provided while working through arthroscopic cannulas and with the use of a knot pusher. At least three RHAPs after placement of most types of sliding or non-sliding knots are necessary for optimal knot integrity.^2^–^6^ Chan et al.^7^ described a technique for switching posts simply by alternating tension on the suture limbs, whereby the knot “flips” and the wrapping limb (or the loop limb) effectively becomes the post. However, Meier et al.^8^ noted that there is a potential flaw when a “flipped” knot is tensioned, past-pointed, or pulled back on by the knot pusher causing the knot to inadvertently revert to its original configuration.

Arthroscopic knot tying requires significant practice and attention to detail, especially in tying the three RHAPs in a knot. A particular technical mistake that has been identified is pulling back the knot pusher either through the arthroscopic cannula or while tying the knot, thereby turning the suture around the post limb and creating unintentional tension applied to the wrapping suture limb. This combination of events “flips” the half-hitch and converts a series of RHAPs into a series of identical half-hitches on the same post, negating the kinking effect created by alternating posts.^8^ Half-hitches tied onto the same post will create insecure knots or suture loops with slippage as the most likely failure mechanism. Chan et al.^5^ evaluated the relative strength of four different stacked half-hitch configurations: identical half-hitches on the same post, reversing half-hitches on the same post, identical half-hitches on alternating posts, and reversing half-hitches on alternating posts. It was determined that the reversing half-hitches on alternating posts are unlikely to fail by slippage, but rather by rupture of the suture material itself.

Suture materials have an effect on the loop/knot security with arthroscopic knots.^9^–^14^,^24^ Herculine™ and Ultrabraid^®^ suture material consists of braided, non-absorbable polyethylene fibers without a longitudinal core, something that is present in FiberWire^®^ and Orthocord^®^. Both Ultrabraid^®^ and Force Fiber^®^ are made with braided UHMWPE with only variations in weaver patterns used. FiberWire^®^ is made of braided polyethylene and polyester fibers coated with a proprietary coating. Orthocord^®^ is made with dyed absorbable polydioxanone core (PDS 68%) with a combination of the undyed, non-absorbable, ultra-high molecular weight polyethylene (UHMWPE 32%) as a sleeve and coated with polyglactin.^15^,^16^ Overall, these sutures are made of similar materials, but with varying designs; thereby different mechanical and handling properties have been reported.

To our knowledge, there has not been a study documenting the effect of different combinations of three stacked half-hitches and suture materials on the loop/knot security of an arthroscopic knot. Testing all combinations systematically would create a large number of possible combinations that would be prohibitively large. It is with this consideration that a reduced number of combinations of three stacked half-hitches were evaluated. The specific aim of this study was to evaluate the effect of different stacked half-hitch configurations and different braided suture materials on an arthroscopic knot’s loop and knot security. The hypothesis was that both stacked half-hitch configurations and specific types of braided suture materials have a significant effect on the knot loop and knot security.

## Materials and Methods

This study design compared three different suture materials tied with five different stacked RHAPs in arthroscopic knots. The three different types of braided materials consisted of the following: Force Fiber^®^ (Stryker, San Jose, CA), FiberWire^®^ (Arthrex, Naples, FL), and Orthocord^®^ (DePuy-Mitek, Warsaw, IN). All arthroscopy suture materials were #2 braided polyblend polyethylene with an estimated length of 48 cm (19 inch) of each material used for tying all of the knots in order for comparison.

**Figure 1:**
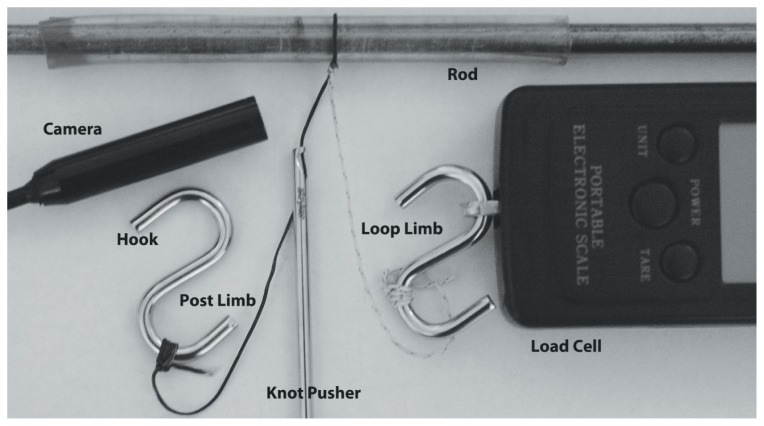
Experimental setup.

**Figure 2:**
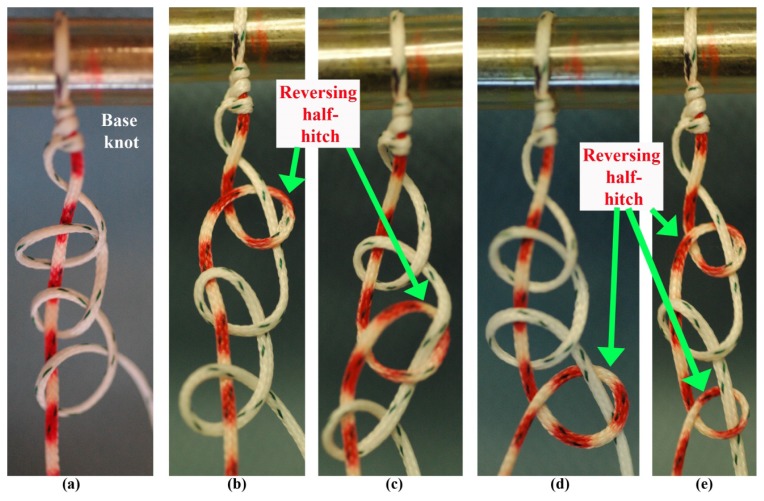
Five different stacked three reversing half-hitches on alternating posts (RHAPs) evaluated. (a) Configuration #1: Identical half-hitches on the same post; (b) Configuration #2: Reversing half-hitch on 1^st^ RHAPs; (c) Configuration #3: Reversing half-hitch on 2^nd^ RHAPs; (d) Configuration #4: Reversing half-hitch on 3^rd^ RHAPs; (e) Configuration #5: Reversing half-hitch on 1^st^ and 3^rd^ RHAPs.

All knots were tied with a standard knot pusher using standard arthroscopic techniques in a dry environment (Figure 1). A load cell was attached to standardize the amount of strength used to tighten the half-hitches. All knot-tying processes in this study began by advancing three identical half-hitches stacked on the same post (base knot) down to a standardized 30 mm circumference post to provide a consistent starting circumference for each knot, as well as replicate the suture loop created during arthroscopic rotator cuff repair. Each suture material was tied with five different stacked knots of three RHAPs with each of these half-hitches tightened manually to at least 45 N using an over-pointing/past-pointing technique. The tightening loads were conformed with the use of a load cell (Protable Electronic Scale, China). The five different stacked RHAPs were as follows: 1) identical half-hitches on the same post (Configuration #1), 2) reversing half-hitch on first RHAPs (Configuration #2), 3) reversing half-hitch on second RHAPs (Configuration #3), 4) reversing half-hitch on third RHAPs (Configuration #4), and 5) reversing half-hitch on first and third RHAPs (Configuration #5; Figure 2). All knots were tied with a knot pusher by the same orthopaedic surgeon. After each knot was tied over the post, the knotted suture loop was removed and trimmed, leaving approximately 6 mm length tags from the most distal end of the knot. Ten knots with each combination of stacked RHAPs knot configuration and each suture material were tied.

**Figure 3:**
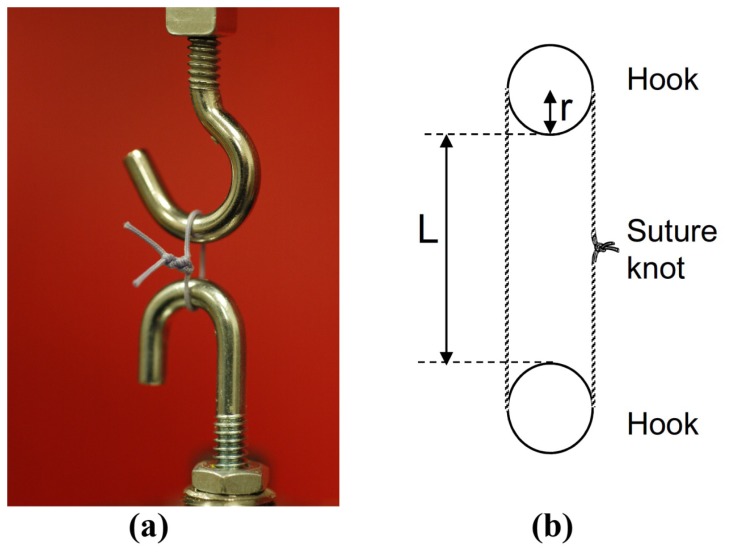
Load-to-failure experimental setup. (a) Experimental setup; and (b) Cross-head displacement measurement.

Servohydraulic Material Testing System instruments (MTS model 810, Eden Prairie, MN) were used to test the knot and loop security of each combination of knots and suture types. Two round hooks with a diameter of 3.9 mm were attached to the actuator and the load cell (Figure 3a). Loops were preloaded to 6 N to avoid potential errors produced from slack in the loops and stretching of the suture materials, as well as providing a well-defined starting point for data recording. The distance between the two rods was measured (cross-head displacement) and the circumference of the loop was calculated according to the formula as:

Eqn 1 CL=(2*L)+(4*r)+Cr

The equation variables are: CL is loop circumference, L is cross-head displacement, r is rod radius, and Cr is rod circumference (Figure 3b).

A single load-to-failure test was performed similar to previously described protocols.^2^,^3^,^13^,^14^,^17^–^19^ Each suture loop was initiated with five preconditioning loading cycles from 6 N to 30 N at 1 Hz. The load was applied continuously at a cross-head speed of 1 mm/sec until complete structure failure. Three millimeters is the point where tissue apposition is lost.^12^,^20^–^22^ Based on this criterion, the current study defined knot slippage of 3 mm (crosshead displacement) as “clinical failure” which is supported by previously performed evaluations of different suture/knot combinations.^2^–^5^,^13^–^15^,^17^–^19^ Load and displacement data were collected at 100 Hz and knot failure mode also was recorded.

## Statistical Analysis

Data retrieved from the load-to-failure tests were analyzed for any differences among sutures, as well as stacked three RHAPs knot configurations using one-way analysis of variance (ANOVA) with the Least Significant Difference (LSD) multiple comparisons post hoc test method in SPSS software (Version 19.0; SPSS, Chicago, IL) with p < 0.05 denoting significant. These analyses were used to determine the statistical relevance of the difference between knot failure load, knot slippage for each suture type, and knot slippage for each knot type. The mean and standard deviation of the ultimate clinical failure load were calculated for each configuration and each type of suture.

## Results

**Figure 4:**
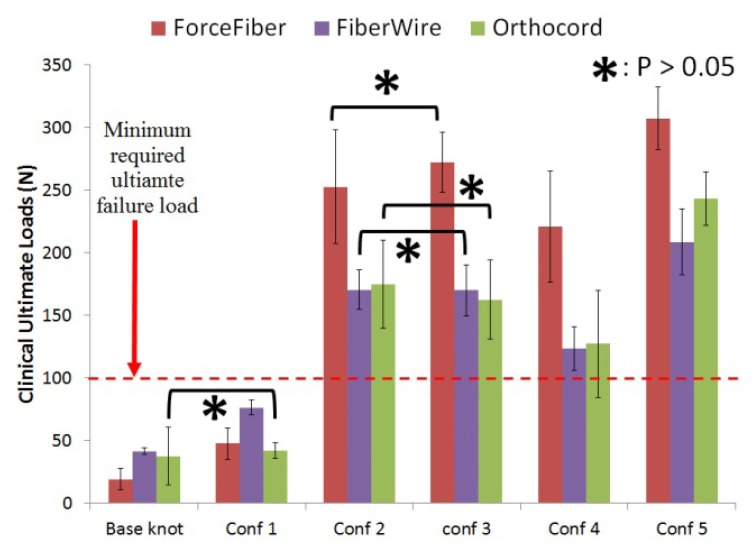
Mean ultimate clinical failure load (3-mm displacement) of sliding knots tied with five different stacked three reversing half-hitches on alternating posts for 3 different braided materials.

Figure 4 shows the mean ultimate clinical failure load (3 mm cross head displacement) of the three different suture materials with five different stacked three reversing half-hitches on alternating posts. In comparison to the base knot (consisting of three identical half-hitches stacked on the same post), knots tied with additional three identical half-hitches stacked on the same post (Configuration #1) using Orthocord^®^ did not show any significant improvement in terms of ultimate clinical failure load (p > 0.05). To the contrary, knots tied with this configuration using Force Fiber^®^ or FiberWire^®^ showed improvement (Force Fiber^®^: 149%; FiberWire^®^: 84%) compared to the base knot, but with an ultimate clinical failure load still less than 100 N. This is critical as 100 N is the estimated minimum required ultimate load per suture during a maximum muscle contraction.^23^

With one of the half-hitches in the RHAPs reversed (Configurations #2 and #3), the mean ultimate clinical failure results showed that there was a significant improvement in knot holding strength compared to Configuration #1 (Force Fiber^®^: 452%, FiberWire^®^: 123%, and Orthocord^®^: 300%), and all knots measured greater than the 100 N failure strength. There were no significant differences detected for knots tied when compared between Force Fiber^®^, Fiberwire^®^, and Orthocord^®^ (p > 0.05). A significant decrease in strength was detected when Configuration #4, where the last half-hitches of the RHAPs were reversed, was compared to Configurations #2 and #3 (Force Fiber^®^: 16%, FiberWire^®^: 28%, Orthocord^®^: 24%).

With two of the half-hitches of the RHAPs reversed (Configurations #5), this knot configuration had a significant improvement in failure strength when compared to Configuration #1 (Force Fiber^®^: 543%, FiberWire^®^: 173%, and Orthocord^®^: 476%). In addition, all knots measured greater than 150 N of mean ultimate clinical failure load. A knot tied with this configuration had improved failure strength significantly when compared to single reversed half-hitches of the RHAPs. This was seen when compared to Configuration #2 (Force Fiber^®^: 22%, FiberWire^®^: 22%, and Orthocord^®^: 39%), Configuration #3 (Force Fiber^®^: 13%, FiberWire^®^: 23%, and Orthocord^®^: 50%), and Configuration #4 (Force Fiber^®^: 39%, FiberWire^®^: 69%, and Orthocord^®^: 91%).

When comparing suture materials, it is observed that for knots tied with at least one half-hitches of the RHAPs reversed, Force Fiber^®^ suture material had a higher mean ultimate clinical failure load than those knots tied with other suture materials (p < 0.05).

**Figure 5:**
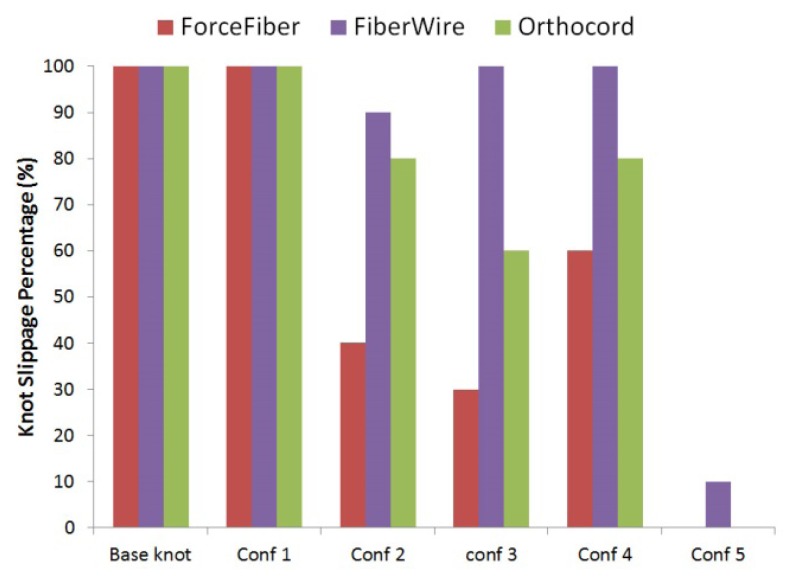
Percentage of knot slipping of knots tied with five different stacked three reversing half-hitches on alternating posts for 3 different braided materials.

Figure 5 shows knot slippage percentage of the knots tied with five different stacked three reversing half-hitches on alternating posts for the three different braided materials. Knots tied with identical half-hitches on the same post (Configurations #1 and #2) resulted in 100% knot slippage. When one of the half-hitches of the RHAPs were reversed (Configurations #2 - 4), there was less chance of knot slippage compared to Configuration #1 (Force Fiber^®^: 30 – 60%, FiberWire^®^: 90 – 100%, and Orthocord^®^: 60 – 80%). The results showed a significant reduction in knot slippage (p < 0.05) when two of the half-hitches of the RHAPs were reversed (Configurations #5). Knots tied with either Force Fiber^®^ or Orthocord^®^ showed no chances of knot slippage, while knots tied with Fiberwire^®^ had about 10% knot slippage.

## Discussion

The results of this study supported our hypothesis that both stacked half-hitch configurations and braided suture materials have a significant effect on the knot loop and knot security. Switching the post limb between throws in a series of half-hitches has increased the knot security by increasing the friction and the internal interference.^2^,^3^,^5^ However, while tying the three RHAPs in a knot, technical errors can occur, such as pulling back the knot pusher while tying the knot or turning the suture around the post limb resulting in an unintentional tension applied to the wrapping limb. These errors can reverse the kinking effect created by alternating posts and result in the incorrect three RHAPs configuration. Errors that occur while attempting to create Configuration #5 can lead to a situation more similar to Configuration #2 or #3. This study was undertaken to determine the effect of three half-hitches of the RHAPs placed after a base knot. The mean ultimate clinical failure strength could be reduced by at least 13% (mean: 40 ± 24%, range: 13% – 91%) if one of the half-hitches was unintentionally “flipped”.

Optimization of knot security for any given knot configuration, suture material, and surgeon experience level during arthroscopic knot tying is crucial.^5^,^12^,^15^–^17^,^23^–^27^ Arthroscopic knot tying requires significant practice and attention to detail especially in tying the three RHAPs in a knot. Therefore, training of arthroscopic knot tying by practicing in a “dry-lab” is recommended. In this study, even with using a load cell to standardize the tightening loads onto each half-hitch, there was an average of 22 N (range: 3 – 45 N) of standard deviation.

Braided non-absorbable polyblend sutures commonly used for arthroscopic knots have better strength profiles and less slippage potential.^2^,^3^,^15^,^17^,^28^–^31^ These studies have evaluated different arthroscopic sliding knot configurations with different suture materials and concluded that a surgeon choosing arthroscopic repair techniques should be aware of the differences in suture material and the variation in knot strength afforded by different knot configurations, as suture material is one of the important aspects of loop security. Our findings are in agreement. Suture materials have a major effect on knot security, especially on a series of three RHAPs, as in theory, these RHAPs should minimize suture friction, internal interference, and slack between loops of the knot, which emphasizes the effect of material selection. Furthermore, our findings also agree with a previous study^14^ that suture materials that have a core in the design (Orthocord^®^, Fiberwire^®^) tend to have the lower ultimate clinical failure strength and higher prevalence of knot slippage compared to the Force Fiber^®^. We suspect that one of the important factors affecting the tendency of knot slippage could be the suture surface characteristics and suture construction.

Our experimental design had certain limitations. First, tying a knot on a standardized rigid smooth aluminum post (30 mm in circumference) differed from what is performed clinically. This setup did not account for the variability seen in clinical practice, especially as the suture loop did not pass through any soft tissue, turn acute angles, risk abrasion on suture anchors, or rub over bony surfaces. Second, the metal hooks used in this study were not compressible and did not interpose in the substance of the knot as soft tissue does in the clinical setting. Third, knots were tied with no tension against the sutures, whereas clinical knots are tied under tension as tissues are pulled together in reconstructions. Fourth, there was no blinding of knot type, and there was no randomization of tying order or testing order. Fifth, only a single load-to-failure test was performed and incremental cyclic loading could be more useful, as it has long been recognized as a leading source of failure in orthopedic repairs. Sixth, all arthroscopic knots were tied with a single knot pusher, whereas in the clinical setting different techniques (e.g., cannula) may result in knots that are not exactly similar to those in the laboratory setting. Seventh, the current study was performed in a dry environment, whereas a fluid environment with varying temperature could affect the effectiveness of knots.

## Conclusions

A significant effect was observed for both stacked half-hitch configuration and suture materials on the knot loop and knot security. Caution should be used when tying the three RHAPs in a knot using standard arthroscopic techniques, a standard knot pusher, and an arthroscopic cannula. This study may provide a solution which potentially could improve the maximum failure loads observed between orthopaedic surgeons, and thereby, achieve better clinical outcomes.
